# Nanoindentation into a bcc high-entropy HfNbTaTiZr alloy—an atomistic study of the effect of short-range order

**DOI:** 10.1038/s41598-024-59761-6

**Published:** 2024-04-20

**Authors:** Iyad Alabd Alhafez, Orlando R. Deluigi, Diego Tramontina, Nina Merkert, Herbert M. Urbassek, Eduardo M. Bringa

**Affiliations:** 1 Institute of Applied Mechanics, Clausthal University of Technology, Adolph-Roemer Str. 2A, 38678 Clausthal-Zellerfeld, Germany; 2 CONICET and Facultad de Ingeniería, Universidad de Mendoza, Mendoza, 5500 Argentina; 3 Physics Department and Research Center OPTIMAS, University of Kaiserslautern-Landau, Erwin-Schrödinger-Straße, 67663 Kaiserslautern, Germany; 4 Centro de Nanotecnología Aplicada, Facultad de Ciencias, Universidad Mayor, Santiago, 8580745 Chile

**Keywords:** Molecular dynamics, Nanoindentation, Dislocations, Plasticity, High-entropy alloys, Theory and computation, Condensed-matter physics

## Abstract

The plastic response of the Senkov HfNbTaTiZr high-entropy alloy is explored by means of simulated nanoindentation tests. Both a random alloy and an alloy with chemical short-range order are investigated and compared to the well understood case of an elementary Ta crystal. Strong differences in the dislocation plasticity between the alloys and the elementary Ta crystal are found. The high-entropy alloys show only little relaxation of the indentation dislocation network after indenter retraction and only negligible dislocation emission into the sample interior. Short-range order—besides making the alloy both stiffer and harder—further increases the size of the plastic zone and the dislocation density there. These features are explained by the slow dislocation migration in these alloys. Also, the short-range-ordered alloy features no twinning plasticity in contrast to the random alloy, while elemental Ta exhibits twinning under high stress but detwins considerably under stress relief. The results are in good qualitative agreement with our current knowledge of plasticity in high-entropy alloys.

## Introduction

The mechanical properties of high-entropy alloys are receiving continuous attention^1–3^ as they combine excellent strength with ductility^4,5^. One of the microscopic origins of their features lies in the slow dislocation activity in these alloys^6,7^ that is caused by the lattice distortion brought about by the mix of atomic constituents with various sizes. Recently, high-entropy alloys forming single-phase bcc crystals have come into the focus of research. Among them, the equiatomic HfNbTaTiZr alloy has become one of the best investigated examples^3^; it is also known as the Senkov alloy^8^. Thus, Xu et al.^9^ directly model the shear strength of the HfNbTaTiZr alloy and observe failure by a bcc-to-fcc phase transformation initiating in HfTi rich regions. Liang et al.^10^ study how variable fractions of Hf and Ta in HfNbTaTiZr alloys affect the tensile strength. Recently, Hf_15_Nb_40_Ta_25_Ti_15_Zr_15_ was found to be both strong and ductile at cryogenic to elevated temperatures^11^.

Due to the chemical complexity of these concentrated alloys, the local stoichiometry may deviate from the overall composition leading to local chemical short-range order. While difficult to monitor experimentally^12^, it is easily accessible to atomistic simulation techniques as emphasized in a recent review article^13^. This short-range order can have a sensitive effect on mechanical properties, as it controls the structure of dislocation nucleation and their mobility^14–18^. Predictions and experiments to taylor yield strength by controlling the short-range order are under way^19^.

The technique of nanoindentation is well known to be able to test mechanical properties under high compressive stress and non-uniaxial load^20^. Molecular dynamics (MD) simulation allows to model the processes occurring under such high loads in detail and has often been employed to study the processes of plasticity, defect formation and even phase transformation occurring^21,22^. Indentation into fcc high-entropy alloys has already been studied in detail previously^23^. Recent MD indentation studies on bcc high-entropy alloys include Ref. 24 which studies a non-equiatomic HfTaTiZr alloy and reports on phase transformation to hcp as the main process of plastic deformation; indeed such phase transformations have been predicted to be relevant for Ti-Zr-(Nb-)Hf-Ta high-entropy alloys on theoretical grounds^25^. Chen et al.^26^ study equiatomic HfNbTaZr alloys which are similar to the Senkov alloy but miss the Ti component; their study reports on the relevance of including short-range order. Liu et al.^27^ investigate a TaTiZrV alloy, albeit with a rather small sample containing less than 1 million atoms; they focus on the initial plasticity and dislocation nucleation processes. In bcc high-entropy alloys, anomalous structures of dislocation cores were identified^28^.

In the present paper, we study the plasticity induced during nanoindentation in the Senkov HfNbTaTiZr high-entropy alloy. Because of the possible influence of chemical short-range order discussed recently^9^, both a random alloy and an alloy with chemical short-range order are considered. In addition, we compare to the well understood case of indentation into an elementary Ta crystal^29–31^, allowing us to highlight the differences in plastic behavior. In particular we can observe the considerably more confined plastic zone in the HEA as compared to an elemental bcc crystal, which gives evidence of the high activation barriers to dislocation motion in these crystals.

## Methods

We study the indentation of a random HfNbTaTiZr alloy; for reference purposes we compare it to that in a pure Ta crystal. For convenience, we will denote the random alloy as the (random) HEA crystal. The single-crystalline HEA substrate has a (100) surface, lateral sizes of 92.5 nm, a depth of 50.6 nm, and contains 21 973 248 atoms. A pure Ta crystal containing the same number of atoms is constructed, which has a lateral extension of 89.8 nm and a depth of 48.9 nm. As it is common in indentation simulations^22^, ideal defect-free crystals are used. Note that – assuming a dislocation density of $10^{12}$ m^-2^ for an annealed metal sample – the dislocation length in the simulation volume is smaller than 1 nm, and hence negligible. From other studies^32^, it is known that quite high initial dislocation densities, in the range of $10^{15}$ m^-2^ and above, would be required to influence the indentation behavior markedly.

In the HEA crystal, the atoms are distributed randomly over the lattice points of a bcc structure with equal fractions of 20%. After relaxation to a final temperature of around 10 mK and low stress components around $10^{-4}$ GPa, it has a lattice constant of 3.3888 Å. This value is in good agreement with experimental data^8,33,34^. For comparison, we note that the lattice constant of Ta in the potential adopted in this study is 3.2897 Å.

The interatomic potential is chosen as an embedded-atom-method-type potential^9^ based on previous work on the construction of alloy potentials^35,36^. Xu et al.^9^ implemented this potential in order to correctly describe the short-range order and the resulting shear strengths in HfNbTaTi-based quinary refractory multi-principal element alloys. We note that an alternative potential for this material is available^37^, but our calculation of elastic constants using that potential does not match experimental results as well as the potential used here, as mentioned by Ref. 9. The potential for the pure Ta crystal has been developed by Ravelo et al.^38^ with a particular focus on describing plasticity and the high-pressure behavior of this metal correctly and has been used previously to model nanoindentation^30,31^.

For the indentation simulation, the bottom and the lateral sides of the samples are fixed in a layer of thickness 6 Å to prevent the whole substrate from any translational movement. The next layers with a thickness of 8 Å are kept at a temperature of 10 mK by a velocity-scaling thermostat^39^. Such a low temperature is chosen to minimize thermal vibrations and help in the identification of defects.

The indenter is modeled as a repulsive sphere. The interaction potential between the indenter and the substrate atoms is limited to distances $r<R$, where *R* is the indenter radius; in this study we fix it to $R = 10$ nm. The indenter interacts with the substrate atoms by the potential

1 $$\begin{aligned} V(r) = \left\{ \begin{array}{ll} k (R-r)^3, &{} r<R, \\ 0, &{} r\ge R, \end{array} \right. \end{aligned}$$

where *r* is the distance of a substrate atom to the center of the indenter. The indenter stiffness has been set to $k= 3.3$ eV/Å^3^.^40,41^

**Figure 1:**
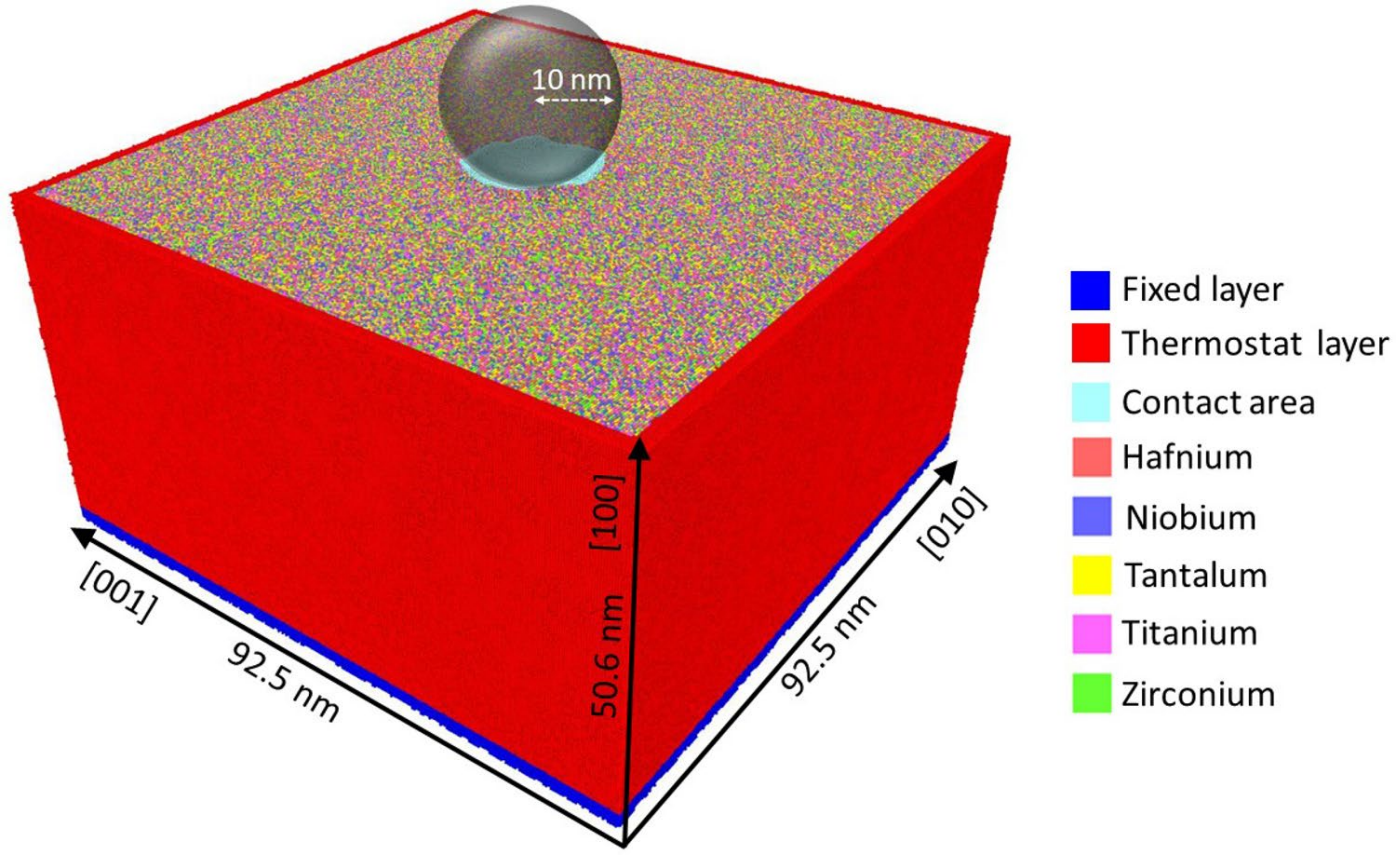
Schematic of the simulation setup. The radius *R* of the indenter and the dimensions of the simulation sample are indicated. The substrate has thermostatting and rigid zones at its boundaries. The contact area at full indentation depth $d=4$ nm, is colored in light blue. .

The indenter is initially positioned immediately above the surface; at time $t=0$ it starts moving perpendicular to the target with a velocity of 20 m/s. We penetrate to a final depth of $d=4$ nm, which is reached at $t=200$ ps. Then the indenter is held fixed for a time of 100 ps, until it recedes with a velocity of 20 m/s. Fig. 1 summarizes the simulation setup as a schematic.

We use the open-source LAMMPS code^42^ with a constant time step of 1 fs to perform the simulations. The free software tool OVITO^43^ is employed to visualize the atomistic configurations. Dislocation profiling has been made by the use of the Crystal Analysis Tool (CAT)^44–46^, including the determination of dislocation lengths and junctions. This tool also has the capability to perform 2D defective structure identification, like twin boundaries. The parameters employed were 0.39 nm for the neighboring cutoff and 0.9 (1.6) nm for the trial and extended circuit lengths, respectively.

### Short-range order

**Figure 2:**
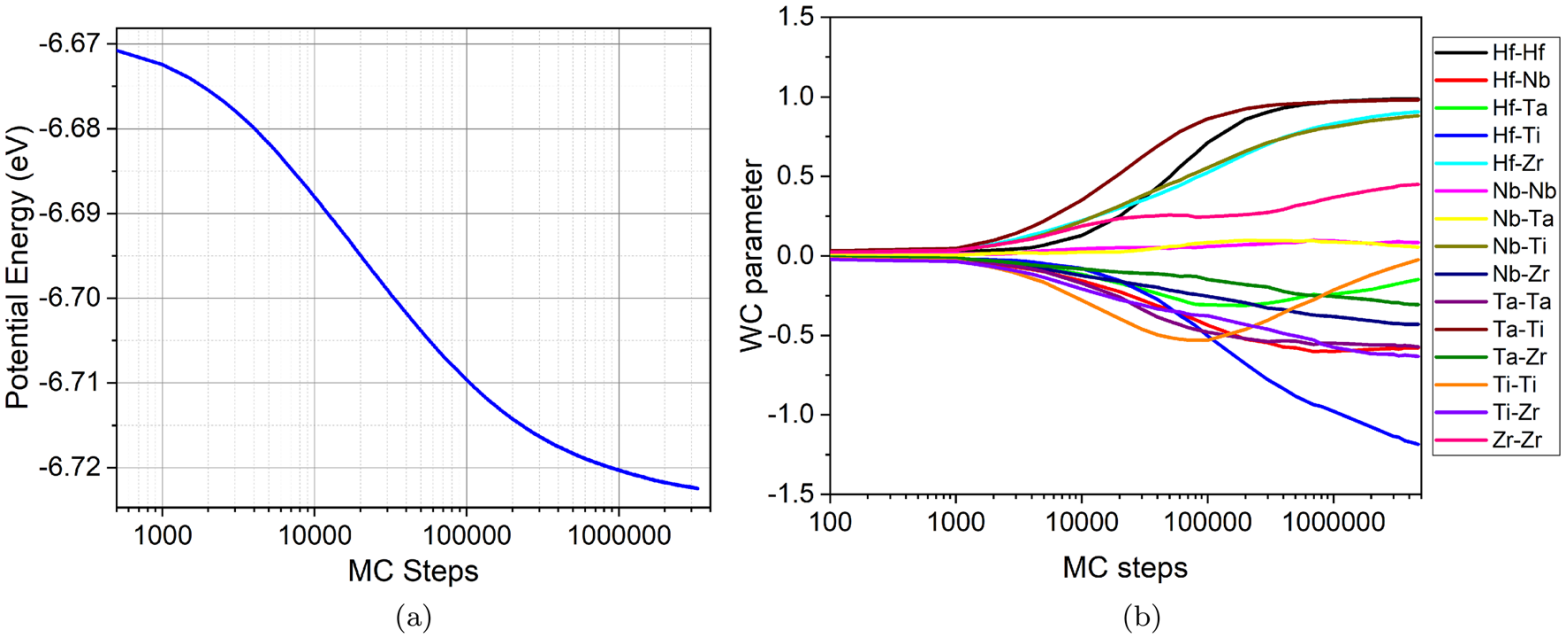
Evolution of the (**a**) potential energy and the (**b**) WC parameters, Eq. 2, during a 4.7-million-step MC run.

Due to the interatomic interactions and to effects during synthesis and thermal processing, alloys may develop chemical short-range order which leads to marked differences in their mechanical behavior, as was for instance demonstrated in Ref. 9 and also for the related HfNbTaZr alloy^26^. We therefore used Monte Carlo simulation at a temperature of 100 K to create an alloy with an equilibrated short-range order. After $4.7 \times 10^6$ Monte-Carlo steps in a sample of $40 \times 40 \times 40$ lattice constants containing 128,000 atoms, a reduction of the potential energy of the sample by around 1% could be achieved, see Fig. 2a.

The established short-range order can be quantified by the so-called Warren-Cowley parameters^47,48^. These are defined as

2 $$\begin{aligned} \alpha _{ij} = 1 - \frac{p_{ij}}{c_j}, \end{aligned}$$

where $p_{ij}$ is the probability of finding an atom of type *j* in the first-neighbor shell of an atom of type *i* and $c_j$ is the (average) concentration of atoms of type *j*. Note that for an equi-atomic alloy, it is $\alpha _{ij} = \alpha _{ji}$, since $p_{ij} = p_{ji}$, and for 5 species the range of $\alpha _{ij}$ is between $-4$ and $+1$. Thus, a random alloy is characterized by $\alpha _{ij} =0$; for $i \ne j$, values of $\alpha _{ij} > 0$ denote atomic repulsion, while $\alpha _{ij} <0$ denotes attraction (ordering, segregation); a random alloy is characterized by $\alpha _{ij} =0$.

**Figure 3:**
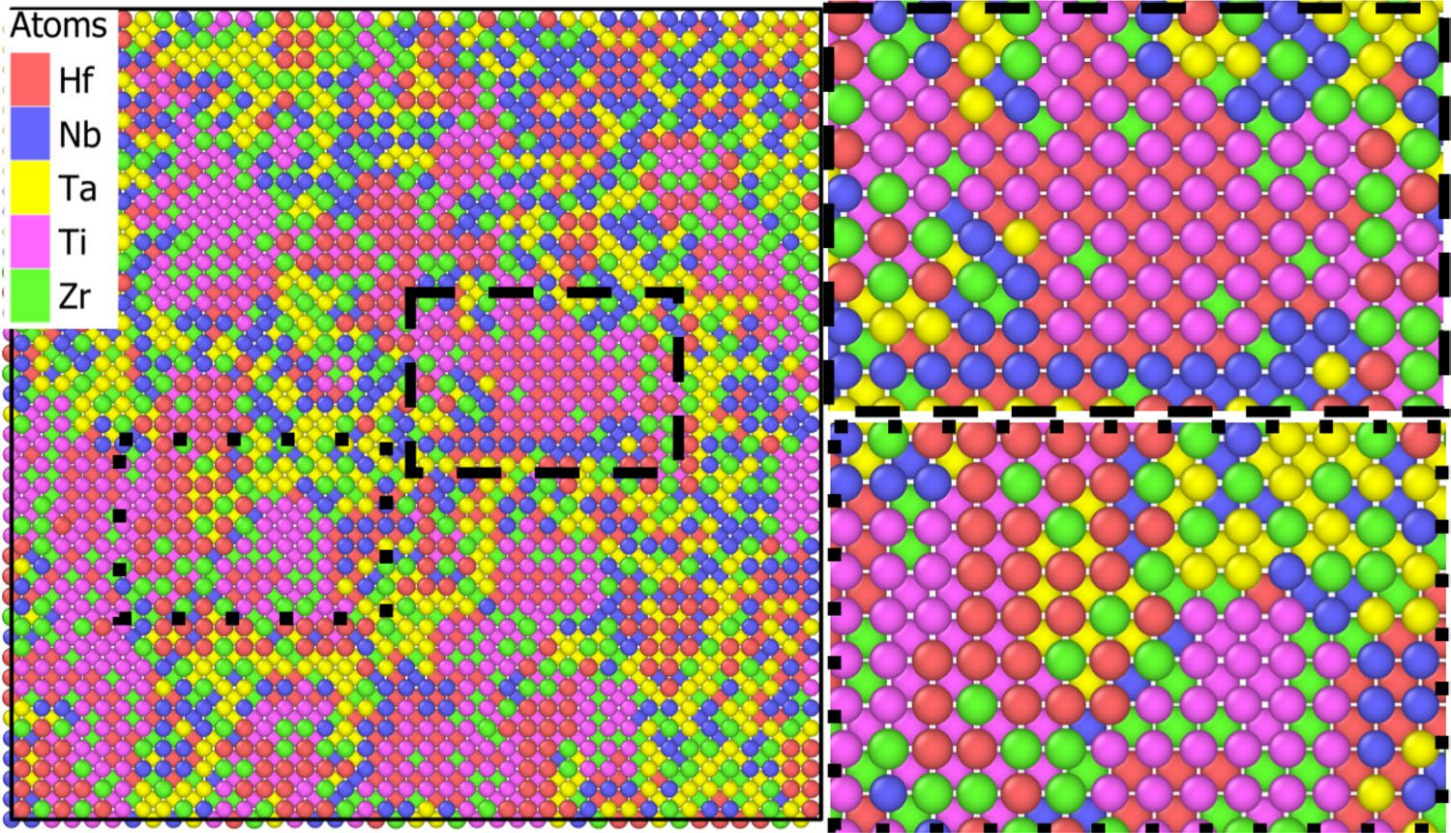
View on a cut through the SRO sample, highlighting atom segregation and formation of B2-ordered regions. Atoms are colored according to atom species.

The evolution of the Warren-Cowley parameters is plotted in Fig. 2b and shows that the chemical evolution reached an equilibrium for most of the species pairs. Thus, for instance, strong tendency of Hf-Ti ordering ($\alpha < -1$) is observed, which is illustrated in Fig. 3; this finding is in agreement with a previous study^9^. A closer analysis shows that small HfTi clusters (precipitates) of a few (2–3) nm diameter with an ordered B2 structure have been established. We note that the presence of such small precipitates has been identified experimentally with electron microscopy, albeit for a different (fcc CoCrNi) alloy^12^. Similarly, but not so pronounced, also TiZr ordering shows up. The large values of the Warren-Cowley parameter for Hf-Hf pairs ($\alpha \sim +1$) demonstrate that Hf prefers not to be nearest neighbor of its own species, but rather second-nearest neighbor such as in the B2 structure. We note that experiments provide a complex view of short-range order in the Senkov alloy. For instance, it has been reported that the alloy decomposes at high temperatures (above 1000 K) into two phases of differing stoichiometry^33,49^.

We conclude that even though atom reordering to induce short-range order does not lead to substantial changes in the potential energy of the sample, it has major consequences for the local alloy structure and may in particular nucleate small clusters of locally ordered regions in the form of B2-ordered precipitates.

For indentation simulations, we construct a sample extending 81.7 nm $\times$ 81.7 nm laterally and 54.4 nm in depth, containing $18.4 \times 10^6$ atoms. For convenience, we shall denote this short-range-ordered HfNbTaTiZr alloy as the SRO sample. Its lattice constant amounts to 3.389 Å, in agreement with the random HEA sample. Indentation on this sample is performed in strict analogy with the random HEA and Ta samples.

## Results

### Indentation force and hardness

**Figure 4:**
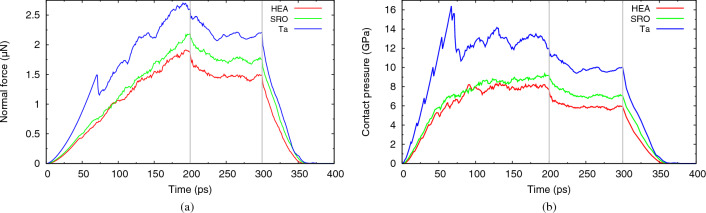
Evolution of the (**a**) normal force and (**b**) contact pressure with time for the random HEA and SRO alloys and the Ta crystal. Indentation proceeds until time 200 ps; the indenter is held constant until 300 ps and then retracted. The vertical lines indicate these times.

The evolution of the indentation force with time is displayed in Fig. 4a. Note that since the indenter moves with a constant velocity of 20 m/s during the indentation and retraction phases, this diagram immediately translates into a force-depth curve. Already during the indentation phase, strong differences between the alloys and elemental Ta show up: For Ta, the elastic phase only ends at 70 ps (depth of 14 Å) and is marked by a strong force drop caused by a burst of dislocation formation^22,30^. The two alloys, on the other hand, end their elastic phase already at 50 ps (depth of 10 Å) showing only a tiny force drop. This indicates that dislocation nucleation occurs more easily in the alloys than in the elemental crystal, but induces less stress relief. This feature also shows up during the later stages of the indentation curve, where force fluctuations induced by dislocation generation lead to strong fluctuations in the force curve for Ta, but not in the alloys.

During the elastic phase, the force follows the Hertzian law,

3 $$\begin{aligned} F = \frac{4}{3} E_{\text{ind}}R^{1/2} d^{3/2}, \end{aligned}$$

as a function of the indentation depth *d*. Since the indenter radius *R* is unchanged, the difference between the curves is entirely caused by the different indentation moduli $E_{\text{ind}}$ which are given by the elastic constants as $E_{\text{ind}}= Y/(1-\nu ^2)$. The elastic constants of the materials studied here are assembled in Table 1. The higher stiffness of Ta explains the steeper force curve in Fig. 4a. The two alloys have quite similar elastic constants; note, however, that SRO is slightly stiffer than the random HEA, explaining the steeper Hertzian rise.

**Table 1:** Elastic constants $C_{ij}$, isotropic elastic moduli and hardness values for the random HEA and SRO alloys and the Ta crystal calculated for the potentials used in this study.

	$C_{11}$	$C_{12}$	$C_{44}$	*B*	*G*	*Y*	$\nu$	$H_{\text{ind}}$	$H_{\text{hold}}$
HEA	156	118	67	131	41	110	0.36	7.9	5.8
SRO	162	120	68	134	43	115	0.36	8.7	7.0
Ta	267	160	86	194	71	190	0.34	12.9	9.8

Data are given in GPa.

*B* bulk modulus, *G* shear modulus determined as a Voigt–Reuss–Hill average, *Y* Young’s modulus, $\nu$ Poisson ratio, $H_{\text{ind}}$ indentation hardness, $H_{\text{hold}}$ hardness in the hold phase.

During the hold phase, the high forces needed to indent to a depth of 4 nm show a considerable decrease, indicating that the material relaxes under the indenter, in particular by dislocation motion and reactions. The force decreases during a time of around 50 ps, indicating that the hold time of 100 ps adopted in our simulations is adequate; note, however, the persistent force fluctuations up to the end of the hold phase indicating that in the strong stress field generated by the indenter, defect processes continue to develop. The final retraction phase is characterized by a monotonic decrease of the force exerted on the receding indenter. The indenter leaves the indent pit at a time of around 360 ps (at a depth of 28 Å) for all three materials, indicating that the indent pit has elastically recovered.

The force *F* can be used to calculate the contact pressure on the indenter via $p=F/A$, see Fig. 4b, where *A* is the contact area projected on the crystal surface. We determined the contact area via the atoms that are in contact with the indenter^50^. After prolonged indentation, the contact pressure stabilizes—apart from fluctuations, which are particularly large for the elemental crystal, Ta, as discussed above. The averaged contact pressure is denoted as the (indentation) hardness $H_{\text{ind}}$, and its value is listed in Table 1. Ta has a considerably larger hardness than the alloys. Interestingly, the SRO alloy shows a by 10 % increased hardness as compared to the random HEA. This is explained by the presence of B2 precipitates that impede dislocation motion and help hardening.

We note that in previous studies, the influence of short-range order on the hardness of the medium-entropy fcc alloy CoCrNi was studied. Experimentally, larger hardness and strength are found for the short-range-ordered alloy than for the random alloy^12^. This fact is corroborated by dedicated simulations of the behavior under nanoindentation^51,52^, where it is found that with increasing short-range order, indentation hardness and strength increase. This strengthening behavior was attributed to an increase of the force needed for dislocation nucleation; also, dislocation pinning was increased by local Ni short-range-ordered structures. The effects found for the fcc alloy thus parallel closely those found in the present study for the bcc HEA.

The dislocation relaxation processes during the hold phase lead to a reduction of the stress field inside the material and hence of the contact pressure. We average the contact pressure over the last 50 ps of the hold phase, after the stress fields stabilized, and denote it as the hold hardness, $H_{\text{hold}}$. Table 1 demonstrates a substantial reduction of the hold hardness with respect to the indentation hardness. Interestingly, this reduction is strongest (27%) for the elementary crystal and least for the SRO alloy (20 %), indicating the enhanced difficulty of dislocation motion and stress relaxation in the alloys. We note that our values of hardness agree extremely well with experimental values between 6 and 8 GPa^53^ and between 4 and 6 GPa^54^, for small penetration depths.

In experiment, indentation hardness is often determined by a method devised by Oliver and Pharr^55–57^. It determines hardness from the force and the geometrically determined projected contact area at maximum load corrected for the sink-in phenomenon; it results in larger values than those listed in Table 1—e.g. $H_{\text{ind}}= 14.5$ for pure Ta—basically because the geometric contact area underestimates the true contact area, which is defined by all atoms in contact with the indenter that bear the load. The method also allows to determine the indentation modulus $E_{\text{ind}}$ from the unloading curve. Again, the values obtained are too high, for instance $E_{\text{ind}}=245$ GPa for Ta, compared to the true value of 215 GPa. This overestimation may be caused by the fact that the unloading curve measures the mechanical properties of a defective and already work-hardened material. We explicitly note that both in the Oliver-Pharr method and in the analysis of the simulation data, pressure is calculated by dividing the normal force by the *projected* area of the indenter. These comments have been made in order to warn against the uncritical use of the Oliver-Pharr method for the analysis of MD data.

### Dislocations

**Figure 5:**
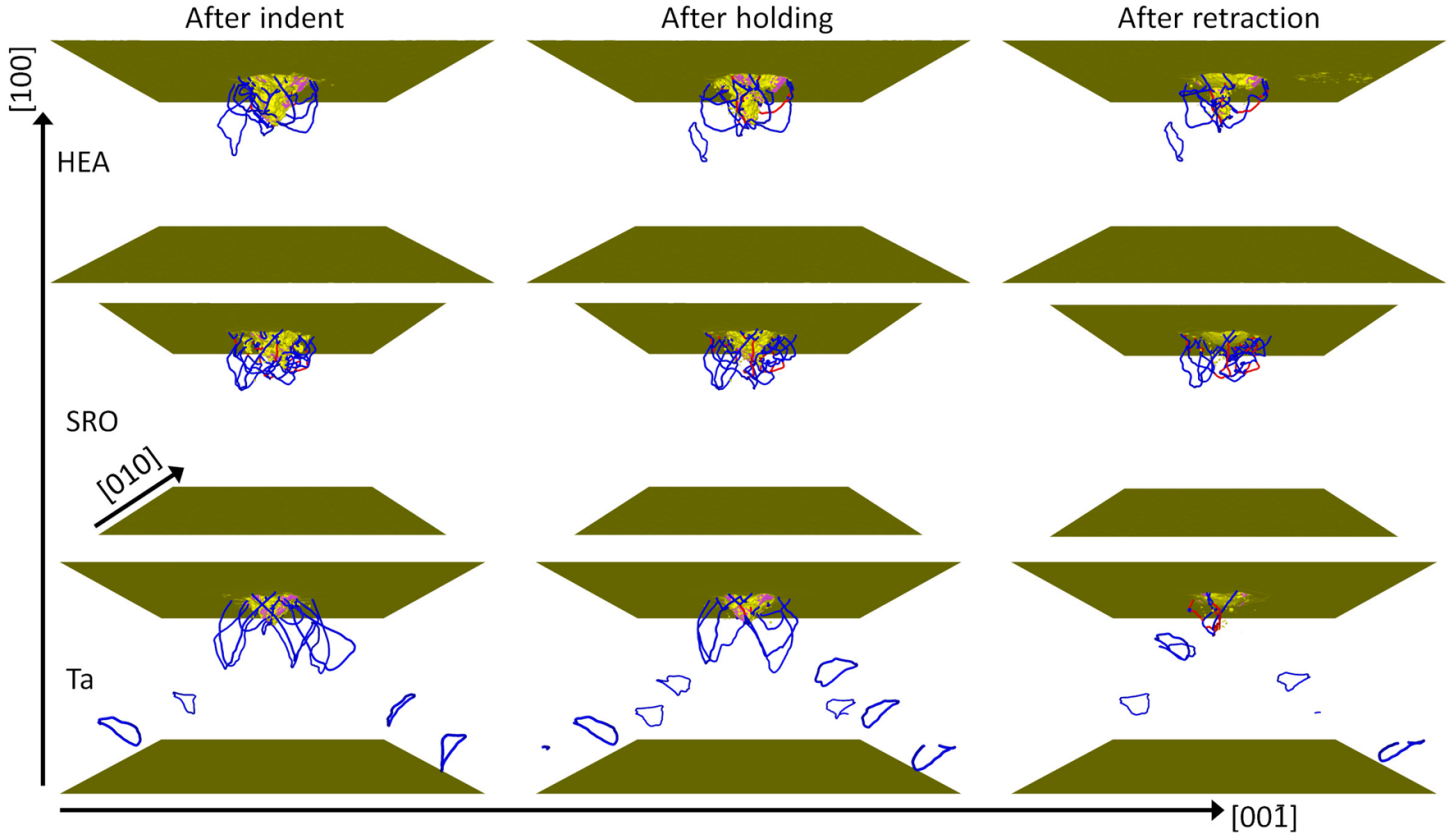
Snapshots showing the dislocation network in the random HEA and SRO alloys and the Ta crystal at full indentation, after the hold phase and after retraction of the indenter. Brown: deformed surface and other unidentified defects. Dislocations are colored according to their Burgers vector $\varvec{b}$: blue $\frac{1}{2} \langle 111 \rangle$, red $\langle 100 \rangle$.

The differences between the forces exerted on the three crystals lead also to considerable differences in the dislocation patterns developing under the indenter. Figure 5 illustrates the dislocation networks established at the end of the indentation and hold phase and also after retraction of the indenter for the three crystals studied. The most noteworthy difference shows up in elementary Ta, in which abundant dislocation emission in the form of prismatic dislocation loops is observed. This phenomenon has been studied in detail in previous simulations^30^; the dislocation loops are formed by the so-called lasso mechanism in which the edge dislocations of extended semiloops approach and annihilate each other such that a full loop is emitted^22,31^. This mechanism is strongly suppressed in the two alloys, since the semiloops do not extend far enough from the indent pit that the two parallel edge dislocations could approach each other. For the random HEA, at least the emission of one small loop can be observed; however, it does not travel far even though the stress field in the vicinity of the indenter is still strong after the hold phase. These snapshots thus demonstrate the small dislocation mobility in the alloys and in particular in the SRO alloy.

A second notable feature relates to the complexity of the network adjacent to the indent pit, which is denoted as the plastic zone. Technically speaking, the extent of the plastic zone is determined by the dislocation segment which is farthest from the indentation point^58^; this distance—denoted as the plastic-zone radius, $R_{\text{pl}}$—is readily determined by the OVITO software^43^ used to analyze the simulation output. Since ejected dislocation loops may move far from the indenter by the stress field exerted by the indenter, they are not included in the definition of the plastic zone. After retraction, only few dislocations remain in the plastic zone of Ta, while the dislocation density in the plastic zone of the alloys remains high. The plastic-zone radius, $R_{\text{pl}}$, may be scaled to the geometric contact radius, $a_c = \sqrt{2Rd - d^2} = 8$ nm, to yield the plastic-zone size factor $f=R_{\text{pl}}/a_c$ in order to allow comparison to other indenter radii *R* and indentation depths *d*. Table 2 assembles these values for the crystals studied here. A clear reduction of the plastic zone during the hold phase for all crystals except the SRO alloy is observed. However, the alloys show only negligible further reduction during the indenter retraction phase while the size of the Ta plastic zone strongly collapses.

**Table 2:** Characteristics of the plastic zone after indent, after hold and after retraction for the random HEA and SRO alloys and the Ta crystal.

	$L_{\text{disl}}$ (nm)	$R_{\text{pl}}$ (nm)	*f*	$\rho$ ($10^{16}$ m^-2^)	*J*	$N_{\text{TB}}$	$N_{\text{twin}}$
After indent							
HEA	455	31.0	3.9	0.366	187	1760	14619
SRO	528	24.6	3.1	1.72	108	736	0
Ta	485	31.9	4.0	0.358	167	1691	4337
After hold							
HEA	398	20.5	2.6	1.12	313	2312	13512
SRO	522	24.3	3.0	1.76	86	596	0
Ta	277	27.1	3.4	0.334	124	1487	3537
After retraction							
HEA	297	19.5	2.4	0.970	160	989	6186
SRO	411	23.3	2.9	1.58	35	0	0
Ta	99	15.4	1.9	0.666	21	292	240

$L_{\text{disl}}$ total dislocation length within plastic zone, $R_{\text{pl}}$ radius of plastic zone, *f* plastic-zone size factor, $\rho$ dislocation density, *J* number of junctions, $N_{\text{TB}}$ number of atoms in twin boundaries, $N_{\text{twin}}$ number of atoms in twinned regions.

These observations on the size of the plastic zone must be complemented by the total length of dislocations, $L_{\text{disl}}$, within this zone, and to the average dislocation density $\rho$, which is determined by $\rho = L_{\text{disl}}/ V_{\text{pl}}$, where $V_{\text{pl}}$ is the volume of the plastic zone. Table 2 shows that the total length of dislocations is comparable after the indent phase, since it is essentially given by the geometrically necessary dislocations which are needed to move the material out of the indent pit into the sample interior^22,59^. However, both the hold and in particular the retraction phase lead to strong reductions of $L_{\text{disl}}$ for the elementary Ta; the reductions for the random HEA are not so pronounced. Thus, the dislocation length is in all phases largest for the SRO alloy, emphasizing the lack of dislocation mobility in this alloy. In the SRO alloy, also the dislocation density is highest; its value stays rather constant at 1.6–$1.8 \times 10^{16}$ m^-2^ in all phases, while the random HEA and Ta have densities below $1 \times 10^{16}$ m^-2^.

**Figure 6:**
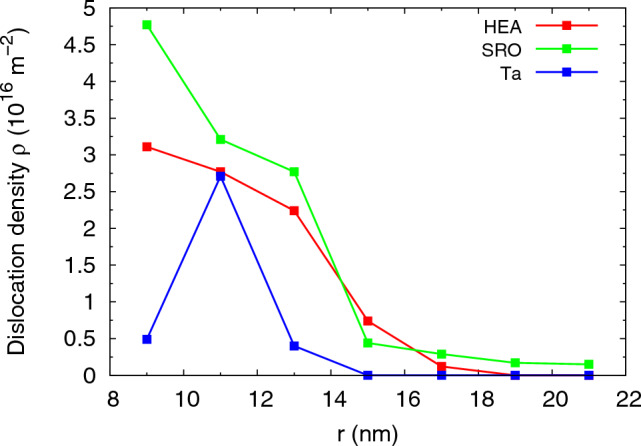
Radial dependence of the dislocation density in the random HEA and SRO alloys and the Ta crystal after retraction.

Figure 6 further illustrates the high density of dislocations by plotting the radial dependence of the dislocation density for the three materials studied after indenter retraction. Clearly, the extreme density values found within the plastic zone strongly exceed the average values given in Table 2. The SRO crystal not only has the highest densities in the plastic zone; also, its plastic zone extends farthest out. This is caused by the fact that 78% of the dislocations generated during the indentation phase survived for the SRO alloy within the plastic zone.

The Dislocation Extraction Algorithm (DXA)^60^ within OVITO also provides the number of dislocation segments in a given frame. After stress release, the average segment length amounts to 4.1 nm in SRO, while it is only 4.7 nm in the random HEA and 5.8 nm in Ta, following the expected trend.

The complexity of the dislocation network can also be assessed by the number of dislocation junctions, *J*. The number of junctions in the elementary crystal is substantially smaller than in the random HEA alloy, indicating the comparably higher network complexity of the alloy visible in Fig. 5. The SRO crystal develops a smaller number of junctions than the random HEA crystal.

**Figure 7:**
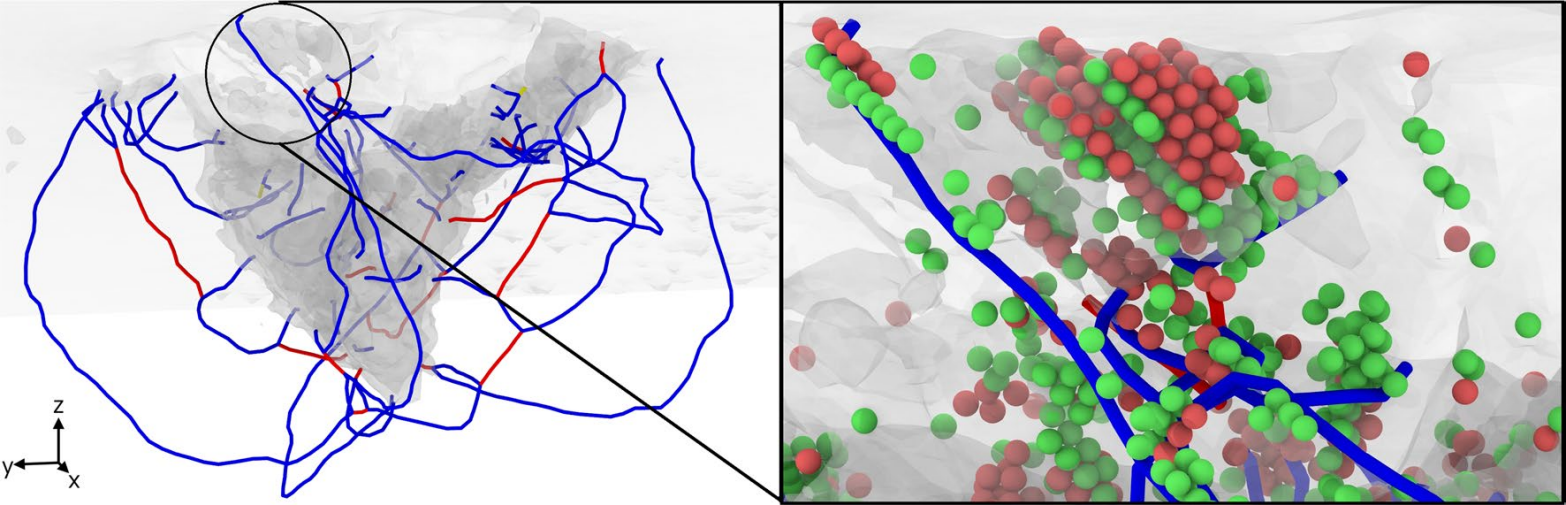
Snapshots showing the dislocation network in the random HEA alloy after the hold phase. The right-hand-side panel zooms into the region highlighted by a circle in the left-hand-side panel. Dislocations are colored according to their Burgers vector $\varvec{b}$ as in Fig. 5, and yellow denotes $\varvec{b}= \langle 110 \rangle$. Atoms are colored according to the local crystal structure: red (hcp) and green (fcc).

Dislocations in bcc crystals have mostly Burgers vector $\varvec{b}= 1/2\langle 111 \rangle$, since these have lowest energy, but under the intense stress fields created during indentation, also dislocations with $\varvec{b}= \langle 100 \rangle$ are observed. We illustrate the network formed by these dislocations in Fig. 7 for the case of the random HEA after the hold phase. The figure illustrates the complexity of the network formed, emphasizing the large number of dislocation junctions present. Dislocations start and end at a highly defective, disordered zone covering the bottom of the indent pit. The analogous snapshot for the SRO is depicted in the Supplementary Material S1 and shows a similarly compact dislocation network.

Table 3 quantifies the fractions of $\varvec{b}= \langle 100 \rangle$ and $\varvec{b}= 1/2\langle 111 \rangle$ dislocations formed and demonstrates the preponderance of the energetically favored $\varvec{b}= 1/2\langle 111 \rangle$ dislocations. In the process of dislocation relaxation after tip retraction, however, dislocation reactions tend to form $\varvec{b}= \langle 100 \rangle$ dislocations, or in other words, a larger percentage of $\varvec{b}= 1/2\langle 111 \rangle$ dislocations annihilate. This occurs in particular in the plastic zone of the elementary crystal, Ta, where most dislocations are annihilated, but to some degree also in the alloy crystals.

**Table 3:** Dislocation types in the simulation volume after indent, after hold and after retraction for the random HEA and SRO alloys and the Ta crystal.

	$\varvec{b}$		Character		
	1/2$\langle 111 \rangle$	$\langle 100 \rangle$	Screw	Edge	Mixed
After indent					
HEA	95	5	37	22	41
SRO	88	12	42	19	39
Ta	98	2	28	42	30
After hold					
HEA	86	14	34	26	40
SRO	87	13	39	23	38
Ta	97	3	17	62	21
After retraction					
HEA	86	14	31	28	41
SRO	83	17	40	24	36
Ta	54	46	10	74	16

Dislocations are categorized according to their Burgers vector $\varvec{b}$—1/2$\langle 111 \rangle$ or $\langle 100 \rangle$—and according to their screw, edge or mixed character. Data are given as fractions (in %) relative to the total length of dislocations.

In elementary bcc metals, screw dislocations dominate the strength since their mobility is small due to the very compact core structure^61^. However, recently Curtin and coworkers emphasized the role that edge dislocations play in the strength of bcc high-entropy alloys^62,63^ and refined theories of the strength of bcc high-entropy alloys based on the different behavior of edge and screw dislocations have been developed^64–66^. We therefore analyzed the dislocations formed in the entire simulation volume according to their screw or edge character. To this end, we determined the angle $\theta$ between the dislocation line and its Burgers vector; if $\theta < 30^\circ$, we count the dislocation as screw, if $\theta > 60^\circ$: as edge, and as mixed in all other cases. Table 3 assembles the fractions of dislocations categorized according to this scheme. For the elementary crystal, Ta, a simple picture shows up: While immediately after indent, all characters show up with similar frequency, after hold and in particular after indenter retraction, most dislocations show edge character; this preponderance of edge dislocations is caused by the ejected prismatic loops which feature a pure edge character and is in agreement with the general knowledge that the mobility of edge dislocations in bcc material by far exceeds that of screw dislocations. This preference of edge dislocations cannot be observed for the alloy crystals. There, a rather uniform mixture of edge and screw character shows up with a small preference, if any, towards screw character.

The limited dislocation mobility in the random HEA severely hinders the dislocation reactions required for prismatic loop emission, e.g. with the lasso mechanism^31^, and leads to the complete absence of prismatic loops for the SRO sample. Something similar was observed under tension for a bulk sample containing a void, where Ta exhibited long dislocation loops while HfNbTaZr showed only short dislocations^67^. We note that experiments, including TEM observations, suggest that the plasticity in HfNbTaTiZr was due to screw dislocation glide^34,68,69^.

In a recent study, Chen et al.^16^ explored the dislocation character created under uniaxial tensile load in a bcc MoTaTiWZr alloy and also report a preponderance of screw over edge dislocations. However, their findings have to be taken with caution, given the small sample sizes used (around $10^5$ atoms).

In nanoindentation experiments, the occurrence of dislocation avalanches has been observed in the Senkov alloy, in particular at higher temperatures^70^. Such avalanches are caused by the sudden release of a group of sluggish dislocations and considerably increase the depth of the plastic zone.

### Other defects and surface imprints

The high stresses in the vicinity of the indenter induce the formation of twin structures in the materials. We identified twin boundaries as well as the volumes of twinned regions. While the analysis of twin boundaries is facilitated by the CAT software^44–46^, the detection of the twinned volumes—i.e., the volume enclosed between twin boundaries—requires the calculation of the misorientation angle of the twinned region with respect to the basic crystal lattice which is accomplished via the Polyhedral Template Matching tool^71^; for further details see Refs. 72 and 73.

**Figure 8:**
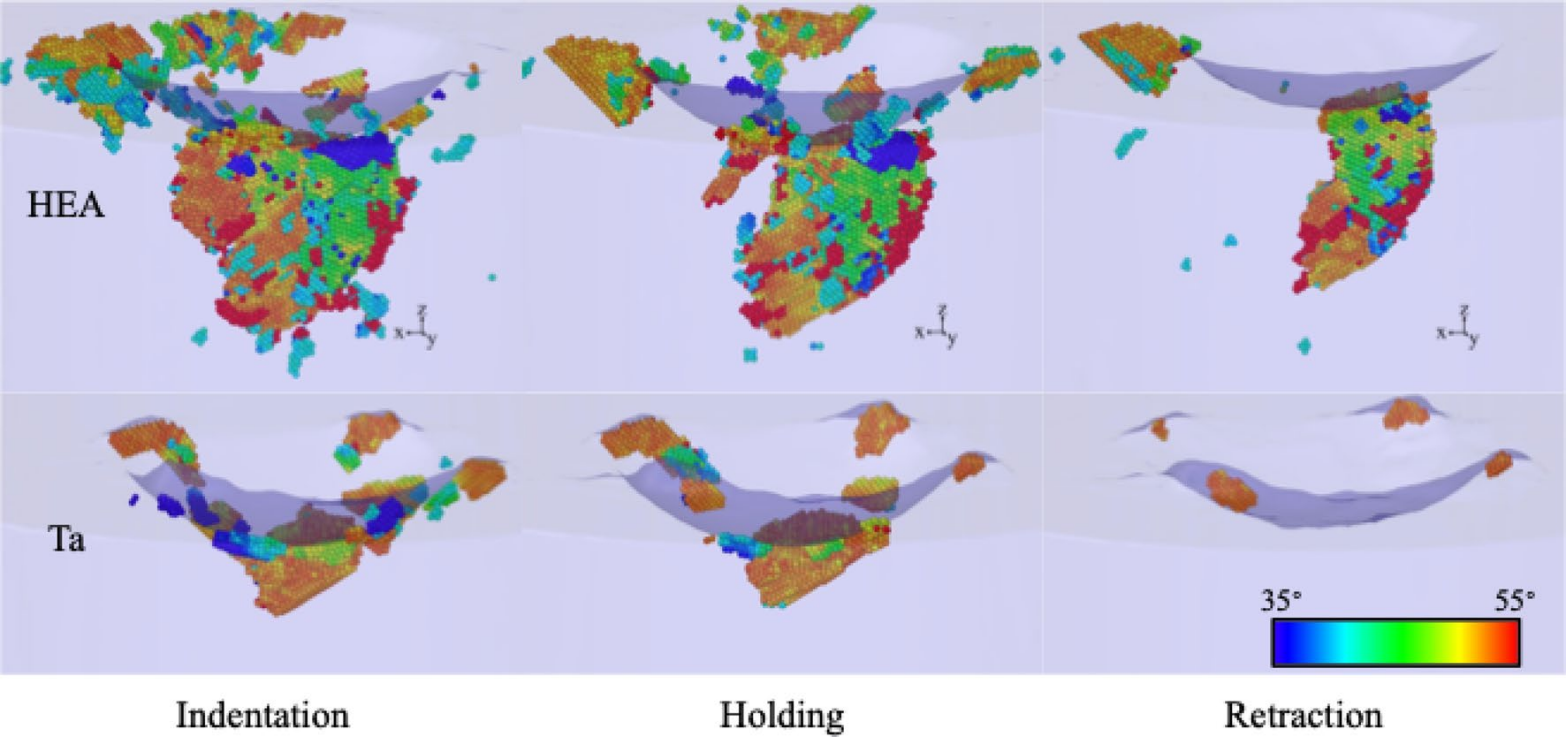
Snapshots showing the twinned regions in Ta and in the random HEA alloy a the end of the indentation, hold and retraction phases. Atoms are colored by the misorientation angle towards the original lattice as given in the color bar. A surface mesh allows to identify the indent pit.

Table 2 assembles the number of atoms present in twin boundaries, $N_{\text{TB}}$, and in twinned regions, $N_{\text{twin}}$, and thus quantifies the amount of twinning occurring in the Ta crystal and in the alloys studied. In addition, Fig. 8 displays the twinned regions in Ta and in the random HEA alloy; as discussed below, such regions do not exist in the SRO alloy. Table 2 shows that Ta features a considerable amount of twinning under stress; both in the indentation and hold phases. The location of these twinned regions is displayed in Fig. 8; the twins are immediately adjacent under the indenter pit as well as in pile-up regions on the surface. After indenter retraction, however, the twins under the indenter re-adjust to the surrounding lattice and only the near-surface twins in the pile-up survive. The importance of detwinning in Ta after stress release has already been demonstrated in previous studies^30,31^.

The random HEA sample, in contrast, shows considerable volume in the twinned regions, extending far below the indent pit; these twins also survive stress release after indenter retraction. This is plausible as it reflects the small mobility of dislocations in this alloy. Note that also in this alloy, at least in one of the surface pile-ups, a twin survives. For the SRO alloy, the amount of twin boundaries detected, is significantly smaller than in the other materials, see Table 2. The twin volume for the SRO sample is actually zero, since the few atoms detected as twin boundaries form small clusters which do not appear to conform with a twin boundary. For this reason, the SRO alloy is not displayed in Fig. 8. The absence of any substantial twinning in the short-range-ordered alloy is plausible as the coordinated crystal rotations required for twinning are thwarted by the breaking of the crystal symmetry caused by the short-range order and the presence of B2 precipitates. This might change for an alloy with larger B2 precipitates.

In experiment,^8,74^ twinning was detected in deformation of HfNbTaTiZr for temperatures lower than 873 K; twinning at large shear strain has also been reported for the same EAM potential used here^9^, and twins under tension were found for simulations of HfNbTaZr when a void was present^67^, and for nanocrystalline samples^72^. However, Hu et al. do not observe twinning under the high applied stress through direct impact Hopkinson bar test and compression strain^75^.

Point defects are created only to a negligible amount in the crystals studied. For the elementary crystal, a few vacancies and interstitial dumbbells are created, and a few vacancies in the alloys. We conclude that creation of point defects does not contribute to the defect formation.

**Figure 9:**
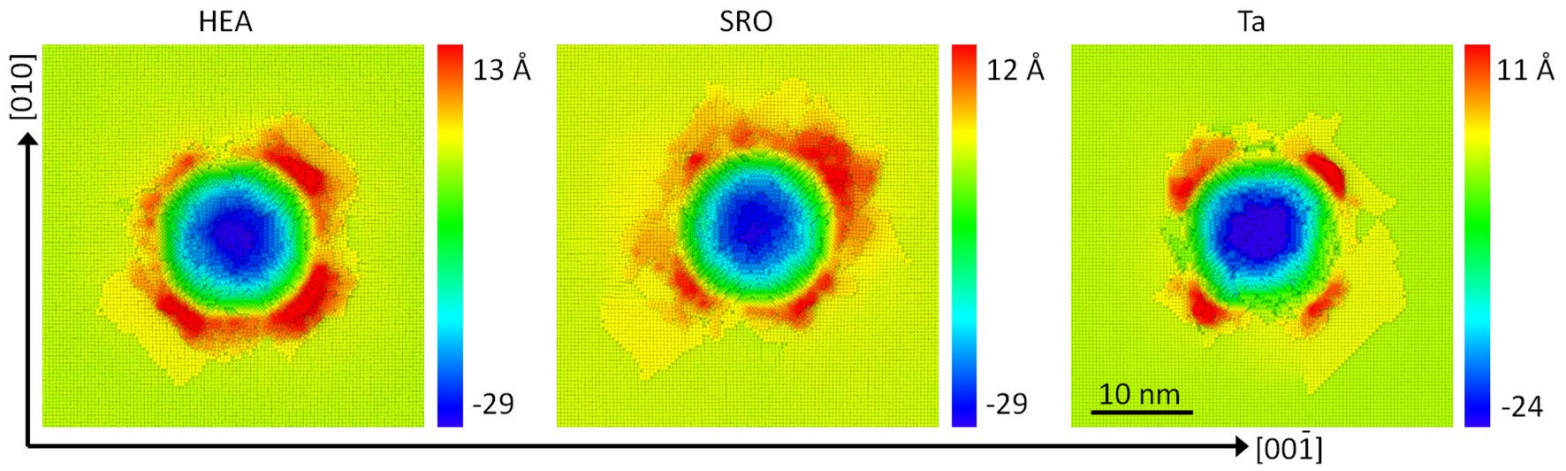
Top view of the indent pits and the surrounding pileups of the random HEA and SRO alloys and the Ta crystal. Color denotes height above the original surface after retraction.

Figure 9 shows the indent imprints and the material pile-up left over on the surface after indenter retraction. Ta features the fourfold symmetry connected to the crystallography of the bcc (100) surface most clearly. The pile-up material is transported on the surface by the activation of the $\{110\}\langle 111 \rangle$ slip system and is therefore situated at $45^\circ$ from the cubic crystal axes on the surface, in agreement with experimental observation^29^. The pile-ups of the alloys exhibit a more circular structure; they also exhibit higher pile-up heights and volume in agreement with the higher dislocation activity in the vicinity of the indenter in these alloys.

### Phase transformation

For the Ta and random HEA samples, we do not find any evidence for phase transformation of the bcc structure to other crystalline forms, with the exception of some hcp and fcc clusters just below the indenter, as shown in Fig. 7. This is in contrast to previous related studies that find large amounts of phase-transformed material^9,24,25^. For the SRO sample, we find large clusters of defective hcp material in the high-stress zone, as seen in the Supporting Material. However, this phase disappears after unloading and we note that other structure detection methods^76^ would be required to better quantify the presence of a phase transformation.

The enthalpy difference between bcc and fcc is typically less than 100 meV/atom for the pressure range under the indenter, as seen in the Supporting Material. For tensile pressure, fcc becomes the preferred phase at $-13$ GPa. For hcp, the enthalpy differences are similar, and hcp becomes preferred at around $-13.5$ GPa. Something similar^67^ happened for a different interatomic potential for HfNbTaZr, with both fcc and hcp becoming preferred over bcc under large tensile stress, but with smaller energy differences, near 0.03 eV. Ref. 67 also found small clusters of fcc and hcp atoms, which were dismissed as detector noise instead of representative of phase change. Simulations^9^ have shown than pure shear of a bulk sample leads to a bcc-to-fcc transition. MD simulations^24^ of nanoindentation of the Ta_0.5_(HfZrTi)_0.5_ metastable alloy showed a bcc-to-hcp transformation. DFT calculations^25^ indicate that an equiatomic alloy, after including lattice relaxation, would be far from the limit of TRansformation-Induced Plasticity (TRIP) behavior due to a bcc-to-hcp ($\omega$ phase) transition, and they reflect on the difficulty in distinguishing a distorted $\omega$ phase from a distorted bcc phase. Experiments^77^ show that TRIP behavior can be obtained by a significant increase of Ti content, compared to equiatomic composition. Finally, Chen et al.^26^ reported indentation-induced amorphization of nanocrystalline HfNbTaZr which, however, vanished after unloading. Inspection of their Fig. 5 indicates growth/decrease in the number of atoms identified by DXA as ‘other’ structures during loading/unloading, but with those atoms having a large degree of order. Therefore, this might only reflect the difficulty in identifying phases under inhomogeneous strain, particularly for the Common Neighbor Analysis (CNA) method used by DXA, and might not represent an actual phase transition. Such misidentification by CNA can occur also for atoms in cubic phases, within the naturally distorted HEA structures^76^. Amorphization, supported by the lack of structure in the pair correlation function, was found in nanocrystalline HfNbTaZr under tension, for 5 nm grain size^72^.

## Summary

We explored the mechanical properties of the high-entropy bcc Senkov alloy by simulated nanoindentation. Both a random HfNbTaTiZr alloy and an alloy exhibiting short-range order were modeled and their performance was compared to that of an elementary Ta crystal. The simulation volume was chosen sufficiently large (> 20 million atoms) that boundary effects were minimized.

Short-range order was introduced into the sample by a Monte Carlo procedure. We observe a slight stiffening of the elastic constants and an increase of the indentation hardness on the 10% level. These changes are caused by the generation of HfTi-rich precipitates in the B2 structure which impede dislocation motion and help hardening. The existence of such precipitates is in line with previous work^9^.

The indentation-induced plasticity is mainly based on dislocation generation and activation. However, dislocation emission from the plastic zone under the indenter, which is a common phenomenon in elemental bcc crystals and also in Ta, is strongly impeded or even absent in the alloys. Also, dislocation recovery after indenter withdrawal is constrained in the alloys; while the radius of the plastic zone in pure Ta shrinks by 50%, it is reduced by only 30 % in the random alloy and stays approximately constant for the short-range-ordered alloy. In consequence, also the dislocation densities in the plastic zone are highest for the alloys, and in particular for the SRO alloy. These features mirror the decreased dislocation mobility in the alloys.

In addition, twinning contributes to the plastic deformation. Twinning is strongest in the random alloy; however, the twinned volume shrinks to one third after unloading. The remaining twins are found both on top of the surface in the pile-up and in the region below the indenter. Also in the elementary Ta crystal, we observe twinning^30,31^; here, however de-twinning is strong such that only < 6% of the twinned volume survives; it is all situated in near-surface pile-up regions. For the SRO alloy, no twinning is found. In that alloy, the breaking of the crystal symmetry and in particular the presence of B2 precipitates prevent the coordinated motion necessary for twin formation, favoring instead some limited transition to the hcp phase, next to the indenter.

In contrast to previous studies^27^, we use samples that are large enough to accommodate the extension of the dislocation network under load including loop ejection. Our comprehensive analysis includes the identification of screw/edge dislocation character as well as the quantification of the twin volumes. In addition, we are the first to compare favorably with experiments regarding both hardness^53,54^ and microstructure^8,34,68,69,74^. Detailed understanding of plasticity might allow design of future alloys with improved performance.

### Supplementary Information

Supplementary Figures.

## Data Availability

All data used for this study are contained in this article.

## References

[1] Zhang Y, Zuo TT, Tang Z, Gao MC, Dahmen KA, Liaw PK, Zhao Ping L (2014). Microstructures and properties of high-entropy alloys. Prog. Mater Sci..

[2] Li Z, Zhao S, Ritchie RO, Meyers MA (2019). Mechanical properties of high-entropy alloys with emphasis on face-centered cubic alloys. Prog. Mater Sci..

[3] George EP, Curtin WA, Tasan CC (2020). High entropy alloys: A focused review of mechanical properties and deformation mechanisms. Acta Mater..

[4] Li Z, Pradeep KG, Deng Y, Raabe D, Tasan CC (2016). Metastable high-entropy dual-phase alloys overcome the strength-ductility trade-off. Nature.

[5] Yang T, Zhao YL, Tong Y, Jiao ZB, Wei J, Cai JX, Han XD, Chen D, Hu A, Kai JJ, Lu K, Liu Y, Liu CT (2018). Multicomponent intermetallic nanoparticles and superb mechanical behaviors of complex alloys. Science.

[6] Wang P, Yuan W, Liu J, Wang H (2017). Impacts of atomic scale lattice distortion on dislocation activity in high-entropy alloys. Extreme Mech. Lett..

[7] Nöhring WG, Curtin WA (2017). Dislocation cross-slip in fcc solid solution alloys. Acta Mater..

[8] Senkov ON, Scott JM, Senkova SV, Miracle DB, Woodward CF (2011). Microstructure and room temperature properties of a high-entropy TaNbHfZrTi alloy. J. Alloy. Compd..

[9] Xu Shuozhi, Jian W-R, Beyerlein IJ (2022). Ideal simple shear strengths of two HfNbTaTi-based quinary refractory multi-principal element alloys. APL Mater..

[10] Liang Z, Wu Y, Miao Y, Pan W, Zhang Y (2023). Composition design and tensile properties of additive manufactured low density Hf-Nb-Ta-Ti-Zr high entropy alloys based on atomic simulations. Materials.

[11] Zhang C, Wang H, Wang X, Tang YT, Qin Yu, Zhu C, Mingjie X, Zhao S, Kou R, Wang X, MacDonald BE, Reed RC, Vecchio KS, Cao P, Rupert TJ, Lavernia EJ (2023). Strong and ductile refractory high-entropy alloys with super formability. Acta Mater..

[12] Zhang R, Zhao S, Ding J, Chong Y, Jia T, Ophus C, Asta M, Ritchie RO, Minor AM (2020). Short-range order and its impact on the CrCoNi medium-entropy alloy. Nature.

[13] Ferrari A, Körmann F, Asta M, Neugebauer J (2023). Simulating short-range order in compositionally complex materials. Nature Comput. Sci..

[14] Chen S, Aitken ZH, Pattamatta S, Zhaoxuan W, Zhi Gen Yu, Banerjee R, Srolovitz DJ, Liaw PK, Zhang Y-W (2021). Chemical-affinity disparity and exclusivity drive atomic segregation, short-range ordering, and cluster formation in high-entropy alloys. Acta Mater..

[15] Sun Z, Shi C, Liu C, Shi H, Zhou J (2022). The effect of short-range order on mechanical properties of high entropy alloy Al_0.3_CoCrFeNi. Mater. Des..

[16] Chen S, Aitken ZH, Pattamatta S, Zhaoxuan W, Zhi Gen Yu, Srolovitz DJ, Liaw PK, Zhang Y-W (2023). Short-range ordering alters the dislocation nucleation and propagation in refractory high-entropy alloys. Mater. Today.

[17] Zhu J, Li D, Zhu L, He X, Sun L (2023). Chemical inhomogeneity from the atomic to the macroscale in multi-principal element alloys: A review of mechanical properties and deformation mechanisms. Metals.

[18] Wang R, Dabo Duan Yu, Tang ZL, Li S, Chen R, Ma C, Yuan W, Bai S, Zhaoping L (2023). Evading dynamic strength and ductility trade-off in a high-entropy alloy via local chemical ordering. Commun. Mater..

[19] Dasari S, Sharma A, Jiang C, Gwalani B, Lin W-C, Lo K-C, Gorsse S, Yeh A-C, Srinivasan SG, Banerjee R (2023). Exceptional enhancement of mechanical properties in high-entropy alloys via thermodynamically guided local chemical ordering. Proc. Natl. Acad. Sci..

[20] Fischer-Cripps, A. C. * Nanoindentation*, 2nd ed. (Springer, New York, 2004)

[21] Ruestes CJ, Alhafez IA, Urbassek HM (2017). Atomistic studies of nanoindentation - a review of recent advances. Crystals.

[22] Ruestes CJ, Bringa EM, Gao Yu, Urbassek HM (2017). “Molecular dynamics modeling of nanoindentation”, in Applied Nanoindentation in Advanced Materials, edited by Atul Tiwari and Sridhar Natarajan (Wiley, Chichester, UK). Chap..

[23] Alhafez IA, Ruestes CJ, Bringa EM, Urbassek HM (2019). Nanoindentation into a high-entropy alloy - an atomistic study. J. Alloy. Compd..

[24] Liu YZ, Sun J, Li HL, Song YY, Hu SP, Song XG, Guo N, Long WM (2023). Molecular dynamics simulations for nanoindentation response of metastable high entropy alloy. J. Mater. Res..

[25] Ikeda Y, Gubaev K, Neugebauer J, Grabowski B, Körmann F (2021). Chemically induced local lattice distortions versus structural phase transformations in compositionally complex alloys. NPJ Compu. Mater..

[26] Chen Y, Reng S, Peng J, Liu X (2023). Chemical short range order and deformation mechanism of a refractory high entropy alloy HfNbTaZr under nanoindentation: An atomistic study. J. Mater. Res. Technol..

[27] Liu X, Hua D, Wang W, Zhou Q, Li S, Shi J, He Y, Wang H (2022). Atomistic understanding of incipient plasticity in bcc refractory high entropy alloys. J. Alloy. Compd..

[28] Zhao L, Zong H, Ding X, Lookman T (2021). Anomalous dislocation core structure in shock compressed bcc high-entropy alloys. Acta Mater..

[29] Biener MM, Biener J, Hodge AM, Hamza AV (2007). Dislocation nucleation in bcc Ta single crystals studied by nanoindentation. Phys. Rev. B.

[30] Ruestes CJ, Stukowski A, Tang Y, Tramontina DR, Erhart P, Remington BA, Urbassek HM, Meyers MA, Bringa EM (2014). Atomistic simulation of tantalum nanoindentation: Effects of indenter diameter, penetration velocity, and interatomic potentials on defect mechanisms and evolution. Mat. Sci. Eng. A.

[31] Remington TP, Ruestes CJ, Bringa EM, Remington BA, Lu CH, Kad B, Meyers MA (2014). Plastic deformation in nanoindentation of tantalum: A new mechanism for prismatic loop formation. Acta Mater..

[32] Alhafez IA, Ruestes CJ, Bringa EM, Urbassek HM (2019). Influence of pre-existing plasticity on nanoindentation - an atomistic analysis of the dislocation fields produced. J. Mech. Phys. Solids.

[33] Malek J, Zyka J, Lukac F, Monika V, Vlasak T, Cizek J, Melikhova O, Adela M, Kim H-S (2019). The effect of processing route on properties of HfNbTaTiZr high entropy alloy. Materials.

[34] Dirras G, Gubicza J, Heczel A, Lilensten L, Couzinié J-P, Perrière L, Guillot I, Hocini A (2015). Microstructural investigation of plastically deformed Ti_20_Zr_20_Hf_20_Nb_20_Ta_20_ high entropy alloy by X-ray diffraction and transmission electron microscopy. Mater. Charact..

[35] Johnson RA (1989). Alloy models with the embedded-atom method. Phys. Rev. B.

[36] Zhou XW, Wadley HNG, Johnson RA, Larson DJ, Tabat N, Cerezo A, Petford-Long AK, Smith GDW, Clifton PH, Martens RL, Kelly TF (2001). Atomic scale structure of sputtered metal multilayers. Acta Mater..

[37] Huang X, Liu L, Duan X, Liao W, Huang J, Sun H, Chunyan Yu (2021). Atomistic simulation of chemical short-range order in HfNbTaZr high entropy alloy based on a newly-developed interatomic potential. Mater. Des..

[38] Ravelo R, Germann TC, Guerrero O, An Q, Holian BL (2013). Shock-induced plasticity in tantalum single crystals: Interatomic potentials and large-scale molecular-dynamics simulations. Phys. Rev. B.

[39] Bussi G, Donadio D, Parrinello M (2007). Canonical sampling through velocity rescaling. J. Chem. Phys..

[40] Kelchner CL, Plimpton SJ, Hamilton JC (1998). Dislocation nucleation and defect structure during surface indentation. Phys. Rev. B.

[41] Ziegenhain G, Hartmaier A, Urbassek HM (2009). Pair vs many-body potentials: Influence on elastic and plastic behavior in nanoindentation of fcc metals. J. Mech. Phys. Sol..

[42] Thompson, A. P., Aktulga, H. M., Berger, R., Bolintineanu, D. S., Brown, W. M., Crozier, P. S., in ’t Veld, P. J., Kohlmeyer, A., Moore, S. G., Nguyen, T. D., Shan, R., Stevens, M. J., Tranchida, J., Trott, C., & Plimpton S. J. LAMMPS—a flexible simulation tool for particle-based materials modeling at the atomic, meso, and continuum scales. *Comput. Phys. Commun.*** 271**, 108171. 10.1016/j.cpc.2021.108171 (2022).

[43] Stukowski, A. Visualization and analysis of atomistic simulation data with OVITO - the Open Visualization Tool. *Model. Simul. Mater. Sci. Eng.* **18**, 015012. 10.1088/0965-0393/18/1/015012. http://www.ovito.org/ (2010).

[44] Stukowski A, Bulatov VV, Arsenlis A (2012). Automated identification and indexing of dislocations in crystal interfaces. Model. Simul. Mater. Sci. Eng..

[45] Stukowski A (2012). Structure identification methods for atomistic simulations of crystalline materials. Model. Simul. Mater. Sci. Eng..

[46] Stukowski A, Arsenlis A (2012). On the elastic-plastic decomposition of crystal deformation at the atomic scale. Model. Simul. Mater. Sci. Eng..

[47] de Fontaine D (1971). The number of independent pair-correlation functions in multicomponent systems. J. Appl. Crystallogr..

[48] Zhang R, Chen Y, Fang Y, Qian Yu (2022). Characterization of chemical local ordering and heterogeneity in high-entropy alloys. MRS Bull..

[49] Schuh B, Völker B, Todt J, Schell N, Perrière L, Li J, Couzinié JP, Hohenwarter A (2018). Thermodynamic instability of a nanocrystalline, single-phase TiZrNbHfTa alloy and its impact on the mechanical properties. Acta Mater..

[50] Ziegenhain G, Urbassek HM, Hartmaier A (2010). Influence of crystal anisotropy on elastic deformation and onset of plasticity in nanoindentation: A simulational study. J. Appl. Phys..

[51] Yang X, Xi Y, He C, Chen H, Zhang X, ShanTung T (2022). Chemical short-range order strengthening mechanism in CoCrNi medium-entropy alloy under nanoindentation. Scripta Mater..

[52] Zhu L, Zhang X, Jian W-R, Xie Z, Yao X (2023). Plastic deformation mechanism and defect patterning under nanoindentation in medium entropy alloy CoCrNi. J. Alloy. Compd..

[53] Sadeghilaridjani M, Pole M, Jha S, Muskeri S, Ghodki N, Mukherjee S (2021). Deformation and tribological behavior of ductile refractory high-entropy alloys. Wear.

[54] Hausild P, Cicek J, Cech J, Zyka J, Kim HS (2020). Indentation size effect in high pressure torsion processed high entropy alloy. Acta Polytechnica.

[55] Oliver WC, Pharr GM (1992). Improved technique for determining hardness and elastic modulus using load and displacement sensing indentation experiments. J. Mater. Res..

[56] Oliver WC, Pharr GM (2004). Measurement of hardness and reduced modulus by instrumented indentation: advances in understanding and refinements to methodology. J. Mater. Res..

[57] Oumarou N, Jehl J.-Ph, Kouitat R, Stempfle Ph (2015). On the variation of mechanical parameters obtained from spherical depth sensing indentation. Int. J. Surf. Sci. Eng..

[58] Gao Y, Ruestes CJ, Tramontina DR, Urbassek HM (2015). Comparative simulation study of the structure of the plastic zone produced by nanoindentation. J. Mech. Phys. Sol..

[59] Swadener JG, George EP, Pharr GM (2002). The correlation of the indentation size effect measured with indenters of various shapes. J. Mech. Phys. Sol..

[60] Alexander Stukowski and Karsten Albe (2010). Extracting dislocations and non-dislocation crystal defects from atomistic simulation data. Model. Simul. Mater. Sci. Eng..

[61] Rodney, D. & Bonneville, J. “Dislocations”, in * Physical Metallurgy*, edited by D. E. Laughlin and K. Hono (Elsevier, 2014) pp. 1591–1680

[62] Maresca F, Curtin WA (2020). Mechanistic origin of high strength in refractory BCC high entropy alloys up to 1900K. Acta Mater..

[63] Baruffi C, Maresca F, Curtin WA (2022). Screw vs. edge dislocation strengthening in body-centered-cubic high entropy alloys and implications for guided alloy design. MRS Commun..

[64] Maresca F, Curtin WA (2020). Theory of screw dislocation strengthening in random BCC alloys from dilute to “high-entropy” alloys. Acta Mater..

[65] Rao SI, Woodward C, Akdim B, Senkov ON, Miracle D (2021). Theory of solid solution strengthening of bcc chemically complex alloys. Acta Mater..

[66] Zhou X, He S, Marian J (2021). Cross-kinks control screw dislocation strength in equiatomic bcc refractory alloys. Acta Mater..

[67] Aquistapace F, Vazquez N, Chiarpotti M, Deluigi O, Ruestes CJ, Bringa EM (2023). Atomistic simulations of ductile failure in a b.c.c. high-entropy alloy. High Entropy Alloys Mater..

[68] Couzinié J-P, Lilensten L, Champion Y, Dirras G, Perrière L, Guillot I (2015). On the room temperature deformation mechanisms of a TiZrHfNbTa refractory high-entropy alloy. Mater. Sci. Eng., A.

[69] Lilensten L, Couzinié J-P, Perrière L, Hocini A, Keller C, Dirras G, Guillot I (2018). Study of a bcc multi-principal element alloy: Tensile and simple shear properties and underlying deformation mechanisms. Acta Mater..

[70] Yin Y-Z, Lu Y, Zhang T-P, Han W-Z (2023). Nanoindentation avalanches and dislocation structures in HfNbTiZr high entropy alloy. Scripta Mater..

[71] Larsen PM, Schmidt S, Schiøtz J (2016). Robust structural identification via polyhedral template matching. Modell. Simul. Mater. Sci. Eng..

[72] Deluigi O, Valencia F, Tramontina DR, Amigo N, Rojas-Nunez J, Bringa EM (2023). Influence of grain size on mechanical properties of a refractory high entropy alloy under uniaxial tension. Crystals.

[73] Alhafez IA, Deluigi OR, Tramontina D, Ruestes CJ, Bringa EM, Urbassek HM (2023). Simulated nanoindentation into single-phase fcc Fe_x_Ni_1-x_ alloys predicts maximum hardness for equiatomic stoichiometry. Sci. Rep..

[74] Senkov ON, Scott JM, Senkova SV, Meisenkothen F, Miracle DB, Woodward CF (2012). Microstructure and elevated temperature properties of a refractory TaNbHfZrTi alloy. J. Mater. Sci..

[75] Hu ML, Song WD, Duan DB, Wu Y (2023). Dynamic behavior and microstructure characterization of TaNbHfZrTi high-entropy alloy at a wide range of strain rates and temperatures. Int. J. Mech. Sci..

[76] Aquistapace F, Amigo N, Troncoso JF, Deluigi O, Bringa EM (2023). MultiSOM: Multi-layer self organizing maps for local structure identification in crystalline structures. Comput. Mater. Sci..

[77] Lilensten L, Couzinié J-P, Bourgon J, Perrière L, Dirras G, Prima F, Guillot I (2017). Design and tensile properties of a bcc Ti-rich high-entropy alloy with transformation-induced plasticity. Mater. Res. Lett..

