# Supplementary material for: Nanoindentation into a bcc high-entropy HfNbTaTiZr alloy—an atomistic study of the effect of short-range order
Source: Sci Rep. 2024 Apr 20;14:9112. doi: 10.1038/s41598-024-59761-6 (PMC11032334; doi:10.1038/s41598-024-59761-6)
Supplement: Supplementary file 1 — Supplementary Figures. [file 41598_2024_59761_MOESM1_ESM.pdf]

Nanoindentation into a bcc high-entropy HfNbTaTiZr alloy – an atomistic study of the effect of short-range order
Supplementary Material

Iyad Alabd Alhafez,[1] Orlando R. Deluigi,[2] Diego Tramontina,[2]
Nina Merkert,[1] Herbert M. Urbassek,[3,*] and Eduardo M. Bringa[2,4]

[1]*Institute of Applied Mechanics, Clausthal University of Technology, Adolph-Roemer Str. 2A, 38678 Clausthal-Zellerfeld, Germany*
[2]*CONICET and Facultad de Ingeniería, Universidad de Mendoza, Mendoza, 5500 Argentina*
[3]*Physics Department and Research Center OPTIMAS, University of Kaiserslautern-Landau, Erwin-Schrödinger-Straße, D-67663 Kaiserslautern, Germany*
[4]*Centro de Nanotecnología Aplicada, Facultad de Ciencias, Universidad Mayor, Santiago, Chile 8580745*

(Dated: September 4, 2023)

Fig. S1 shows the SRO sample after the indentation phase. We observe a phase transition from bcc to hcp near the indentation zone: some hcp clusters present twins as fcc phase. To prove that these are hcp clusters, in Fig. S2 we filter out atoms with coordination less than 12.

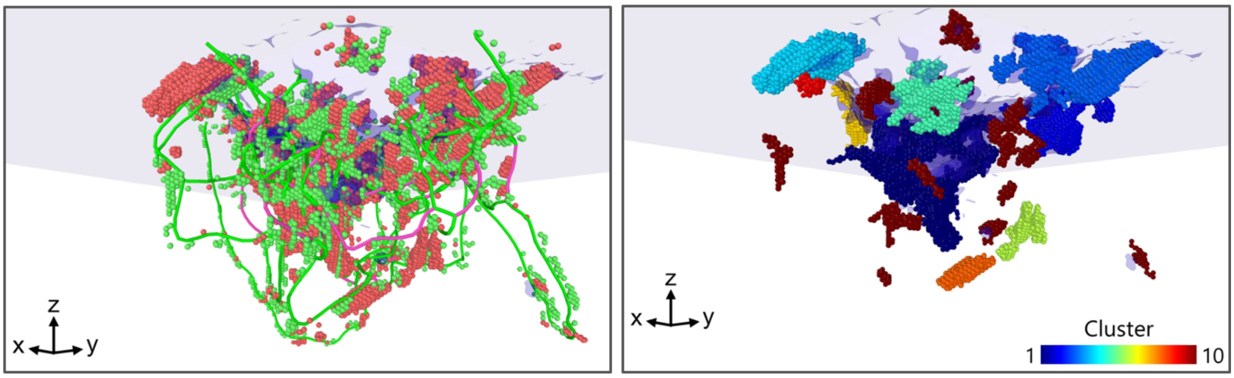

(a) (b)

FIG. S1: Snapshot of the SRO alloy after indent. Only hcp and fcc atoms are shown, with structure given by PTM (rmsd=0.1). A surface mesh is also shown. (a) Atoms colored by PTM structure: red (hcp) and green (fcc), and dislocations from DXA, colored according to their Burgers vector: green $\boldsymbol{b} = 1/2\langle 111\rangle$ and purple $\boldsymbol{b} = \langle 100\rangle$. (b) Atoms colored according to cluster affiliation; the color codes enumerates the clusters according to their size. Only clusters containing more than 30 atoms are shown.

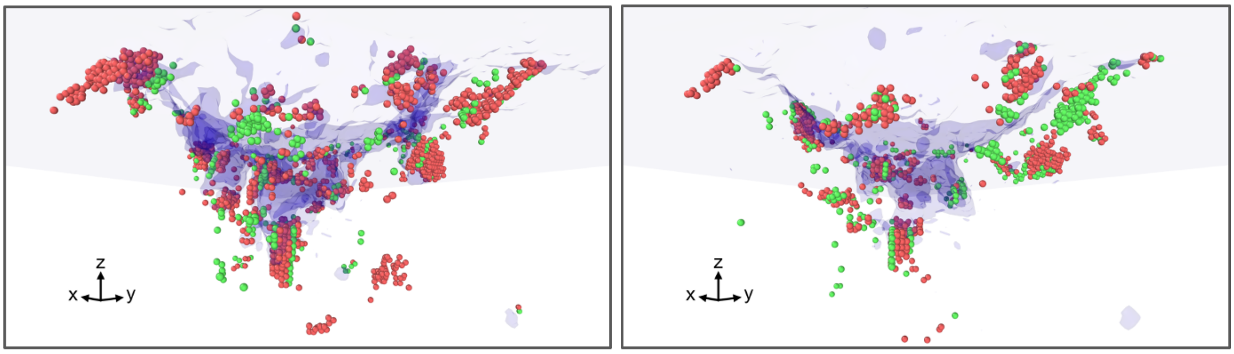

(a) (b)

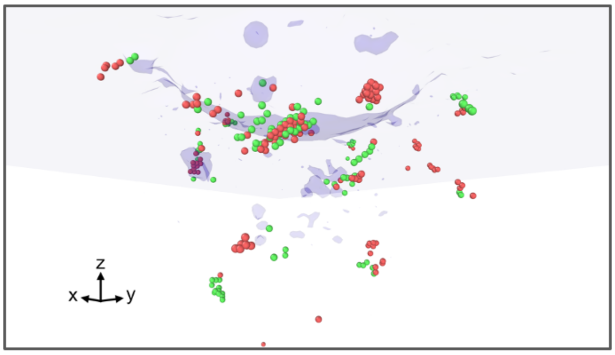

(c)

FIG. S2: Snapshot of the SRO alloy after (a) indent, (b) hold and (c) retraction. Only hcp and fcc atoms are shown, with structure given by PTM (rmsd=0.1). A surface mesh is also shown. Only atoms with coordination 12 are shown. The lack of large 12-coordinated clusters after the indent stage signals negligible phase change.

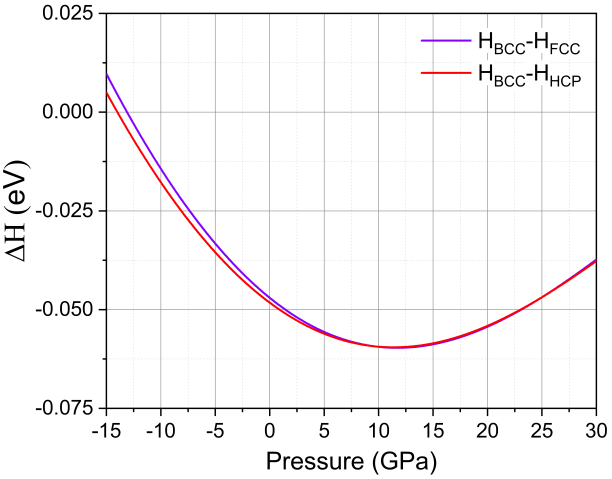

FIG. S3: Enthalpy difference of bcc, hcp and fcc phases in the random HEA alloy as a function of hydrostatic pressure. The plot demonstrates that close-packed structures become stable only for tensile pressures exceeding around 12 GPa, but not for compressive pressures in the range occurring under indentation.
